# A Three-Step System to Ensure Correct Attribution of Named Clinicians for Inpatients

**DOI:** 10.7759/cureus.26347

**Published:** 2022-06-26

**Authors:** Abdel Saed

**Affiliations:** 1 Trauma and Orthopaedics, Royal Victoria Hospital, Belfast, GBR

**Keywords:** communication, trauma and orthopaedics, quality improvement, named consultant, responsible clinician

## Abstract

Background: The Francis report recommends that all patients admitted into a UK hospital must have a named identifiable and suitably trained consultant or clinician in charge of their care. This is regarded as a shared responsibility as highlighted by the recommendations made by the General Medical Council Best Practice guidance. However, this can become more error-prone, particularly in acute trauma and orthopaedic inpatients when the named consultant may change numerous times.

Methods: We conducted an audit reviewing all the inpatients in the acute trauma and orthopaedic wards and then reaudited twice following the introduction of the three-step system. The results were then analysed and compared with previous cycle results.

Results: Initially following the introduction of the three-step system, there were poorer outcomes. Inpatients with the correct named consultant declined from 47% to 37%. However, following further education and training of each respective member of the multidisciplinary roles, the results were much improved with 88.9% of the inpatients having the correct named consultant.

Conclusions: Ensuring that all inpatients have the correct named consultant is a shared responsibility amongst all health and social care staff involved with the patient. This audit highlights that attributing specific roles to relevant members of the multidisciplinary team can improve communications and patient care.

## Introduction

Following the adverse events at the Mid Staffordshire National Health Service (NHS) Foundation Trust, the Francis report was released. One of the vital recommendations was that all patients admitted into a UK hospital must have a named identifiable and suitable trained consultant or clinician in charge of their care [1]. General Medical Council (GMC) Best Practice highlights that every member of the multidisciplinary team ‘must contribute to the safe transfer of patients between healthcare providers and between health and social care providers.’ This includes sharing all the relevant clinical and non-clinical information with the clinician taking over the care and ensuring the named clinician or team has accepted responsibility when the role of the previous team or clinician has ended. This is especially important for vulnerable patients or patients who may have capacity impairments [2-7].

The GMC guidance for doctors acting as responsible consultants or clinicians also supports these recommendations, and highlights adhering to this will contribute to ensuring care is properly coordinated and will also allow for patients and individuals close to the patient to be able to communicate effectively with the responsible clinician and team should they have any questions or concerns [8].

This is further supported by the Academy of Medical Royal Colleges which has released guidance highlighting the responsibilities and roles of responsible clinicians or consultants.

These include the following:

- Ensuring full coordination of the patient’s hospital stay.

- Ensuring that all inpatients and individuals close to them are aware of who has overall responsibility for their care.

- Where appropriate, responsibility is transferred to another responsible and suitably trained consultant or clinician [9-12].

It is this final point that this study was most interested in. Particularly in an acute Trauma & Orthopaedic ward, the named clinician can change numerous times depending on the policy of the respective trust. The admitting consultant, the consultant leading the post-take ward round, and the operating consultant may all be different. Therefore, the main aim of this audit was to see if we were adhering to the Gold standard guidelines as per the GMC and ensuring that all inpatients have the correct identifiable named clinician in charge of their care [2].

## Materials and methods

At our trust, we did a retrospective audit cycle to assess the accuracy of responsible named clinical for acute fracture inpatients.

Initially, we did a snapshot audit of all acute trauma and orthopaedic inpatients on the 19th of May 2019. We compared the consultant named on the ward dashboard which is used to locate all patients on the ward along with their named clinician and then compared this with their documentation. Depending on where each patient was metaphorically along their inpatient stay meant that the named consultant for each patient may have been, the consultant on call on the day of admission, the consultant dictating on the post-take ward round or the operating consultant (if the patient had an operation.)

The data were collected and entered into an excel sheet and the results of the first cycle were presented in the monthly audit meeting in the presence of consultants, registrars, and other members of the Multidisciplinary Team. PowerPoint was used to present the findings and feedback was received from the attendees following the presentation. Suggestions on possible changes to improve the results were discussed and noted.

The changes agreed upon and disseminated to the entire team via email and the upcoming audit meeting were a three-step system that included:

1) A copy of the on-call rota would be left on each of the trauma and orthopaedic inpatient wards to allow overnight staff to be able to identify correct consultants when phoning through patient admission.

2) Each patient on the admission sheet will have the initials of the consultant who will conduct the post-take ward round for them. This will be done by the trauma coordinator and then the copy of this list will be handed over to the fracture clinic nurse in charge and the sisters of each ward at the bed meeting.

3) The ward clerks will be informed by the ward sister of each patient and who the responsible consultant is. The ward clerk will also be responsible for reviewing all-day-1 post-operative patients and changing the named consultant accordingly.

Two further re-look audit cycles were conducted on the 25th of July 2019 and the 25th of March 2021 once interventions were established and implemented.

## Results

The first cycle included 89 patients across four inpatient trauma and orthopaedic wards.

Initial results were adverse, 42 patients (47%) were under the correct consultant and the majority, 46 patients (52%), were identified as having the incorrectly named consultant assigned to their care. There was also one patient (1%) who was not admitted into the hospital system despite being an inpatient for over 24 hours. The reasons for this are unclear.

Following these findings, it was decided, with the approval of the senior team, that a three-step system plan would be actioned to improve the outcomes.

A subsequent re-audit was conducted on the 25th of July 2019 which found that results had poorer outcomes compared with the initial cycle. Thirty-three patients (37%) were under the correct consultant and 55 patients (62%) were under the incorrectly named consultant. Again, there was one patient who was not admitted into the system despite being in hospital for 24 hours.

**Figure 1:**
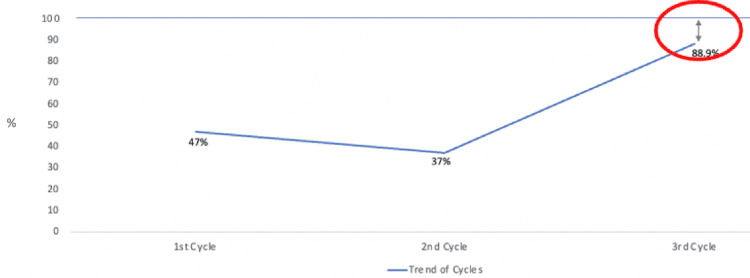
Trend of Audit Results

This was followed by further education and a reminder of the three-step system. The goal of the second cycle was for further education and training to be put in place to ensure there was no confusion as to what each relevant member of the multidisciplinary team’s role was. This was specifically in the form of informal reminders and discussions had with each relevant professional regarding their roles in the process. A further audit was conducted on the 25th of March 2021. This time the results were much improved. There were 66 inpatients across the four inpatient wards. Slightly reduced capacity was due to the unprecedented COVID-19 pandemic percussions, therefore, necessitating social distancing. However, 58 patients (89.9%) were under the correct named consultant with only eight patients (11.1%) under the incorrect consultant. All patients were admitted to the system within 24 hours of hospital admission (Trend of audits shown in Figure 1).

## Discussion

The study has demonstrated the need for awareness and training in ensuring all respective members of the multidisciplinary team are aware of their roles in ensuring all patients have the correct named clinician throughout their inpatient stay. We identified a drawback in our practice, in which we found the majority of patients had the incorrectly named consultant during their inpatient stay and was therefore not in accordance with the GMC best practice and Academy of Royal Medical Colleges guidelines [8]. Therefore, we realised that we needed a plan that was more robust than education alone. Although initial results had shown poorer outcomes compared with the initial audit, after further education and training it was pleasing to see the results had improved significantly following the third cycle. Our results show that our interventions have improved the quality of care overall [13].

Our audit has demonstrated that by reviewing our practices regularly and distributing the results, we were able to improve our service without requiring the use of a significant number of extra resources or increasing costs.

Having the incorrectly named consultant is not only a communication problem for the patient and the multidisciplinary team but it can also result in adverse outcomes, delay in treatment, morbidity and mortality, and medicolegal implications [14,15].

There are however limitations that need to be disclosed. The numbers of patients included are small, and we have yet to include outlier patient data, and a closer look is needed to evaluate weekend data. Furthermore, although our findings showed significant improvement following the introduction and reinforcement of the change, there was still 11.1% of patients under the wrong consultant (circled in red in Figure 1). Subsequently, we can conclude that the intervention we made, although positive, was not wholly embedded with all the medical staff it was intended for. The allocation of roles in the three-step system process may not have been effectively disseminated to all those involved including staff that fills in for potential absentees.

## Conclusions

It was clear to see that the generic shared responsibility highlighted by GMC best practice and the Academy of Medical Royal Colleges were ineffective in our trust. Our approach to introducing a three-step system with specific members of the multidisciplinary team having clear roles was in an effort to reduce the number of inpatients with the incorrectly named consultant. Although initial results were ineffective, it was clear to see that following time, further support, and reinforcement of the planned three-step system results were much improved.

Although ensuring the named consultant for each patient is a multidisciplinary shared process, there is evidence that significant improvements were made in our trust when roles were attributed to specific members of the team.
